# The Macrophage-Specific Promoter *mfap4* Allows Live, Long-Term Analysis of Macrophage Behavior during Mycobacterial Infection in Zebrafish

**DOI:** 10.1371/journal.pone.0138949

**Published:** 2015-10-07

**Authors:** Eric M. Walton, Mark R. Cronan, Rebecca W. Beerman, David M. Tobin

**Affiliations:** 1 Department of Molecular Genetics and Microbiology, Duke University Medical Center, Durham, North Carolina, United States of America; 2 Department of Immunology, Duke University Medical Center, Durham, North Carolina, United States of America; 3 Center for Host-Microbial Interactions, Duke University Medical Center, Durham, North Carolina, United States of America; National University of Singapore, SINGAPORE

## Abstract

Transgenic labeling of innate immune cell lineages within the larval zebrafish allows for real-time, *in vivo* analyses of microbial pathogenesis within a vertebrate host. To date, labeling of zebrafish macrophages has been relatively limited, with the most specific expression coming from the *mpeg1* promoter. However, *mpeg1* transcription at both endogenous and transgenic loci becomes attenuated in the presence of intracellular pathogens, including *Salmonella typhimurium* and *Mycobacterium marinum*. Here, we describe *mfap4* as a macrophage-specific promoter capable of producing transgenic lines in which transgene expression within larval macrophages remains stable throughout several days of infection. Additionally, we have developed a novel macrophage-specific Cre transgenic line under the control of *mfap4*, enabling macrophage-specific expression using existing floxed transgenic lines. These tools enrich the repertoire of transgenic lines and promoters available for studying zebrafish macrophage dynamics during infection and inflammation and add flexibility to the design of future macrophage-specific transgenic lines.

## Introduction

The zebrafish has been widely adopted in the study of myriad vertebrate biological processes, including inflammation and infection. Two important features of zebrafish as a model system are the optical transparency of embryos and larvae and the ease with which transgenesis can be accomplished [1–5]. In simple fluorescent reporter zebrafish lines, cell-type specific fluorescent reporter expression combined with the optical transparency of the larvae allows for real-time visualization of specific cell lineages [6–10].

Transgenic lines involving the labeling or manipulation of immune cell lineages can offer an unprecedented view into the dynamics of the vertebrate innate immune system and its interactions with a number of human pathogens [11]. The larval zebrafish has been used to study a growing array of bacterial pathogens, including *Mycobacterium* [12–19], *Pseudomonas* [20–22], *Salmonella* [23, 24], and *Listeria* spp. [25, 26]. Studies of fungal disease pathogenesis, including infections with *Candida albicans* [27] and *Aspergillus fumigatus* [28] have utilized the zebrafish as a model host, as have investigations of Influenza A virus and Chikungunya virus [29, 30]. These infection studies benefit from the ability to visualize host-microbe interactions within a transparent and genetically tractable vertebrate host that can be produced in large numbers.

The larval zebrafish possesses an innate immune system that comprises cell types common to all vertebrate innate immune systems, including those of mammals [31, 32]. This similarity has allowed for the study of host-pathogen interactions with high spatial and temporal resolution, and has been facilitated by the use of transgenic lines with fluorescently labeled innate immune cell types [6, 7, 9, 33]. Macrophages and neutrophils are among the first immune cell lineages to encounter invading pathogens and are particularly well conserved between zebrafish and mammals in both cellular composition and function [34, 35].

However, more than 15 years after the first descriptions of zebrafish macrophages [35, 36], there exist a limited number of transgenic tools for visualizing and manipulating macrophages in live zebrafish. Transgenic lines have been generated using the myeloid differentiation factor *pu*.*1* which labels macrophages, neutrophils and their precursors [31]. Further analysis of the role of *pu*.*1* in zebrafish myelopoiesis identified four putative macrophage-specific genetic markers, all of which exhibited striking transcriptional downregulation upon knockdown of *pu*.*1* [37]. One such marker, *mpeg1*, was later identified as macrophage specific and transgenes were constructed under the control of upstream regulatory elements of the *mpeg1* locus [6]. Two additional transgenic lines have been generated that drive transgene expression in macrophages: one operates under the control of the *immunoresponsive gene 1 (irg1)* promoter and labels macrophages only upon activation [10]; the other is a bacterial artificial chromosome (BAC) transgenic utilizing the zebrafish *macrophage colony stimulating factor* (*fms*) promoter that labels both macrophages and skin xanthophores [38]. However, transgenic lines based on *mpeg1* have to date remained the primary tool for real-time, *in vivo* analyses of global zebrafish macrophage dynamics [34, 39].

Downregulation of endogenous zebrafish *mpeg1* expression during infection with *M*. *marinum* or *S*. *typhimurium* has been previously described [16], and our lab has observed severe attenuation of *mpeg1* transgene expression in larval macrophages occurring as early as three days post-infection with *M*. *marinum*. We describe the development of a new macrophage-specific *mfap4* transgenic line that, in contrast to *mpeg1*, remains stable during mycobacterial infection. In addition, we describe the generation of a macrophage-specific Cre transgenic line that will allow lineage-tracing and easy combinatorial transgenic approaches to investigating *in vivo*, real-time macrophage dynamics.

## Materials and Methods

### Zebrafish handling

Animal research and protocols were approved by the Duke University IACUC A145-14-06. Adult zebrafish were housed under standard conditions using an automated system for water changes and maintenance of pH and conductivity (Aquaneering). Larvae were raised at 28.5°C in incubators. Manipulation and imaging were carried out using tricaine anesthesia as described below. Euthanasia of any severely ill or moribund animals was performed by immersion in water at a temperature below 4°C, as approved by IACUC.

### Generation of the *mfap4* promoter element

The forward and reverse primers (5’-CATGTTCTCGAGGCGTTTCTTGGTACAGCTGG–3’ and 5’-CATGTTGGATCCCACGATCTAAAGTCATGAAGAAAGA–3’, respectively) were used to amplify a 1.6 kb region immediately upstream of the third codon of the second exon within the *mfap4* locus. The BAC clone CH73-131D4 obtained from Children’s Hospital Oakland Research Institute (CHORI) was used as the PCR template. The linear *mfap4* promoter amplicon was cloned into the p5E-MCS vector of the Gateway Cloning system via restriction digest and ligation with XhoI/BamHI and T4 DNA Ligase (NEB) as described previously in [14].

### Start codon mutagenesis

The p5E-*mfap4* Entry Clone was linearized and amplified via inverse PCR using the following primers: 5’-CTTCTCACTCTCTCCTCAACAG–3’ and 5’-GTAAGTTCTGTGGCTGTTTTATTC–3’. The start codon was mutated from ATG to ATT via Gibson Assembly using the linearized backbone and the following single-stranded DNA oligonucleotide: 5’-CTGAGCTGTTGAGGAGAGAGTGAGAAGATTGCAGTAAGTTCTGTGGCTGTTTTATTCC–3’ as described previously in [14].

### Construction of the *mfap4*:*tdTomato* transgene

The tdTomato fluorescent protein was amplified from pTEC5 (a kind gift of Dr. Lalita Ramakrishnan, University of Cambridge) using the following primers: Forward- 5’-GGGGACAAGTTTGTACAAAAAAGCAGGCTGGACCATGGTGAGCAAGGGCGAGGAG–3’ and Reverse–5’-GGGGACCACTTTGTACAAGAAAGCTGGGTTTACTTGTACAGCTCGTCCAT–3’. Amplified tdTomato sequence was BP cloned into pDONR221 to generate pME-tdTomato.

The complete transgene was subsequently generated by recombination of *p5E-mfap4*, *pME-tdTomato*, and *p3E-polyA* [4] elements into the destination vector *pDestTol2pA2* using the LR Clonase II Plus enzyme mix, resulting in *pDestTol2; mfap4*:*tdTomato*.

### Construction of the *mfap4*:*tdTomato-CAAX* transgene

*pME-tdTomato-CAAX* was generated as described [14].

The complete transgene was subsequently generated by recombination of *p5E-mfap4*, *pME-tdTomato-CAAX*, and *p3E-polyA* elements into the destination vector *pDestTol2UbpA* using the LR Clonase II Plus enzyme mix, resulting in *pDestTol2; mfap4*:*tdTomato-CAAX*.

### Construction of the *mfap4*:*Turquoise2* transgene

Both pME-Turquiose2 and the complete *pDestTol2; mfap4*:*Turquoise2* were generated as described [14].

### Construction of the *mfap4*:*iCre*:*p2A-tdTomato* transgene

The *mfap4*:*iCre*:*p2A-tdTomato* transgene was generated through multisite gateway recombination of *p5E-mfap4*, *pME-iCre*, and *p3E-p2A-tdTomato* into *pDestTol2UbpA*. *pME-iCre* was generated by amplifying the codon-optimized iCre from pDIRE (Addgene plasmid 26745 from Zeller lab, [40]) with attB1 and attB2 flanked primers: Forward– 5’-GGGGACAAGTTTGTACAAAAAAGCAGGCTCCACCATGGTGCCCAAGAAGAAGAG–3’, Reverse– 5’-GGGGACCACTTTGTACAAGAAAGCTGGGTCGTCCCCATCCTCGAGC–3’ and recombining with pDONR 221 by BP recombination (Life Technologies).

To generate *p3E-p2a-tdTomato*, tdTomato was amplified from pTEC5 with the forward primer 5’-GGGGACAGCTTTCTTGTACAAAGTGGTTGGATCCTTCAGTCTCGAGATGGTGAGCAAGGGCGAGGAG–3’ and reverse primer 5’-GGGGACAACTTTGTATAATAAAGTTGTTACTTGTACAGCTCGTCCAT–3’ and gateway cloned in a BP cloning reaction into pDONR p2R-P3. The resulting plasmid contained BamHI and XhoI restriction sites upstream of the Tomato start codon. The *p3E-Tomato* construct was digested with BamHI and XhoI and annealed linkers encoding the porcine teschovirus–1 2A sequence (p2A) [41] were ligated into the plasmid to generate *p3E p2A-tdTomato*. Annealed linker sequences encoding p2A were as follows: Top strand– 5’- GATCCGGAAGCGGAGCTACTAACTTCAGCCTGCTGAAGCAGGCTGGAGACGTGGAGGAGAACCCTGGACCTC–3’ and bottom strand– 5’- TCGAGAGGTCCAGGGTTCTCCTCCACGTCTCCAGCCTGCTTCAGCAGGCTGAAGTTAGTAGCTCCGCTTCCG–3’.

pDestTol2UbpA was generated by amplifying the ubiquitin B poly A signal with the following primers– 5’-TATTATACATAGTTGATATTCTCAGTATCCCCTGCTTA–3’ and 5’-CGACGGCCAGTGAATTATACTTCTCATTTCGCATCTTAT–3’; and amplifying the pDestTol2pA2 backbone with the following primers– 5’-AATTCACTGGCCGTCG–3’ and 5’-ATCAACTATGTATAATAAAGTTGAACGAG–3’, and joining the PCR products with Infusion (Clontech).

The complete transgene was subsequently generated by recombination of *p5E-mfap4*, *pME-iCre*, and *p3E-p2A-tdTomato* elements into the destination vector *pDestTol2UbpA* using the LR Clonase II Plus enzyme mix, resulting in *pDestTol2; mfap4*:*iCre*:*p2A-tdTomato*.

### Construction of *mfap4*:*dLanYFP-CAAX* transgene

For *Tg(mfap4*:*dLanYFP-CAAX)* ^*xt11*^ only, the *p5E-mfap4* Entry Clone was slightly modified by the inclusion of the p2A sequence at the 3’ end of the promoter element, in-frame with both the upstream *mfap4* coding sequence and downstream transgene coding sequence. This sequence was included as an additional mechanism to ensure that any translation of the *mfap4* coding sequence would be separated from the transgene polypeptide. No differences in expression were observed between this line and other *mfap4* lines. The modified *p5E-mfap4* was generated by inverse PCR of *p5E-mfap4* using the primers 5’-GGCTGAAGTTAGTAGCTCCGCTTCCCACGATCTAAAGTCATGAAG–3’ and 5’-AGACGTGGAGGAGAACCCTGGACCTGGATCCACTAGTTCTAGAGCGG–3’, followed by Gibson Assembly (NEB) of the linearized *p5E-mfap4* amplicon and a single-stranded DNA oligonucleotide 5’- AGCGGAGCTACTAACTTCAGCCTGCTGAAGCAGGCTGGAGACGTGGAGGAGAACCCTGGA–3’.

The sequence of dLanYFP was PCR amplified from pNCS dLanYFP (Allele Biotechnology) with the primers 5’-GGGGACAAGTTTGTACAAAAAAGCAGGCTGGACCATGGTGAGCAAGGGCGAGGAG–3’ and 5’-GGGGACCACTTTGTACAAGAAAGCTGGGTAGATCTACTTGTAGAGCTCGTCCATGCCG–3’. These primers resulted in a silent SacI site in the 3’ end of the LanYFP coding sequence and a BglII site downstream of the dLanYFP stop codon. The PCR product was cloned by BP gateway cloning (Life Technologies) into pDONR221 to generate pME-dLanYFP. The resulting pME-dLanYFP construct was digested with SacI and BglII and annealed oligos encoding the human H-Ras prenylation signal [42, 43] were cloned into the plasmid to generate pME-dLanYFP-CAAX. Oligo sequences for the linker are: top strand- 5’-CTACAAGAAGCTGAACCCTCCTGATGAGAGTGGCCCCGGCTGCATGAGCTGCAAGTGTGTGCTCTCCTA–3’, bottom strand- 5’-GATCTAGGAGAGCACACACTTGCAGCTCATGCAGCCGGGGCCACTCTCATCAGGAGGGTTCAGCTTCTTGTAGAGCT–3’.

The complete transgene was subsequently generated by recombination of the modified *p5E-mfap4*, *pME-dLanYFP-CAAX*, and *p3E-polyA* elements into the destination vector *pDestTol2pA2* using the LR Clonase II Plus enzyme mix, resulting in *pDestTol2; mfap4*:*dLanYFP-CAAX*.

### Generation of transgenic zebrafish lines

In all cases, transgenic lines were generated as follows: Single-cell zebrafish embryos were injected with approximately 1nl of a mixture containing 50ng/μl of transgenic construct (i.e., *pDestTol2; mfap4*:*dLanYFP-CAAX*), 25ng/μl of Tol2 transposase mRNA, and 0.17% phenol red in 1x Buffer Tango. The Tol2 transposase mRNA was generated via *in vitro* transcription from T3TS-Tol2 [2] using the mMessage mMachine T3 Kit (Life Technologies). Larvae positive for transgenesis were selected at 48 hours post-fertilization on the basis of detection of transgene fluorescence. Adults from these founder populations were individually outcrossed to wildtype fish, and larvae positive for macrophage fluorescence were retained as F1 populations.

### *M*. *marinum* larval infections, imaging, and quantitation

The transgenic zebrafish lines *Tg(mpeg1*:*tdTomato-CAAX)* ^*xt3*^ or *Tg(mfap4*:*dLanYFP-CAAX)* ^*xt11*^ were crossed to generate double transgenic larvae. Larvae positive for both red and yellow fluorescence were anesthetized in 160 μg/ml Tricaine (MS–222, Sigma Aldrich) and injected via the caudal vein at 48 hours post-fertilization with 100–200 *M*. *marinum* expressing the Cerulean fluorescent protein. Larvae were imaged at 1 day post-infection and 5 days post-infection in all three fluorescent channels as well as transmitted light. All imaging for this experiment was carried out using a Zeiss Observer Z1 inverted microscope. Quantitation of fluorescence was carried out using Zen Image Processing software (Zeiss).

Confocal images were acquired on a Leica SP8 Confocal Microscope using a 25x water-immersion lens.

### *M*. *marinum* adult infections and frozen tissue sections

Adult infections, tissue preparation, and frozen sectioning were performed as described in [14].

## Results

### Construction of macrophage-specific transgenic lines based on *mfap4*.

**Fig 1:**
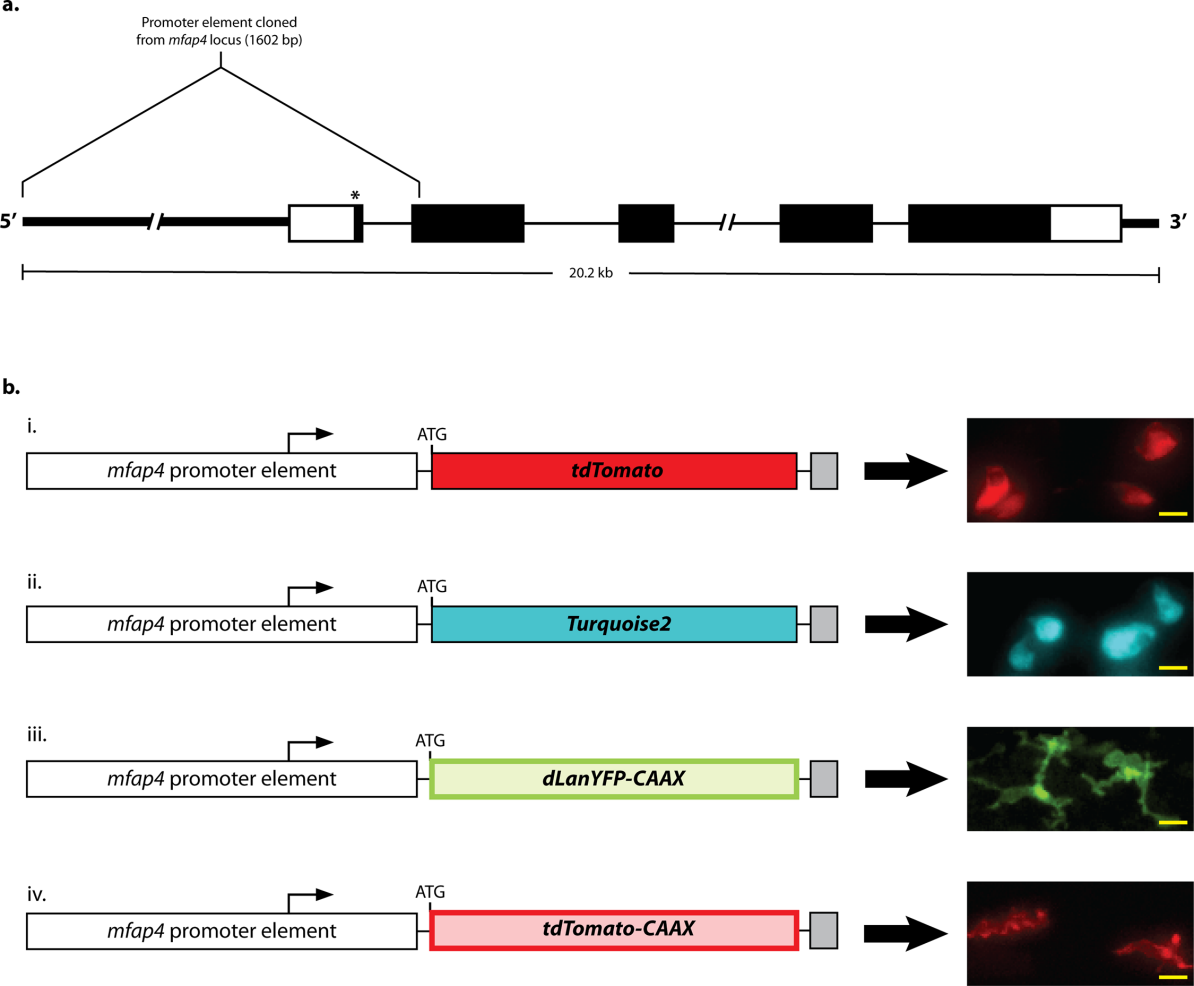
*mfap4* transgene design. **a.** Schematic of the *mfap4* promoter sequence with respect to the full endogenous *mfap4* locus. The full promoter element spans 1,602 bp. Thick lines represent genomic sequence flanking the 5’ and 3’ UTRs. Empty and solid boxes represent UTRs and coding regions, respectively. Thin lines represent introns. The asterisk denotes the location of the start codon mutation (AT**G**>AT**T**). **b.** Schematics of *mfap4* transgenes and examples of their resulting expression. The bent arrows mark transcription start sites; “ATG” marks the beginning of the fluorescent protein ORF. White boxes depict the *mfap4* promoter as the 5’ Element of an expression construct generated via the Gateway Recombination System; colored boxes depict Middle Elements containing fluorescent protein ORFs; grey boxes depict 3’ Elements containing a poly-adenylation signal. The lines connecting each element represent small linker sequences present as a result of the recombination process. i,ii. Expression of cytosolic tdTomato and Turquoise2 fluorescent proteins, respectively. iii,iv. Expression of membrane localized dLanYFP and tdTomato, respectively. The addition of the CAAX prenylation motif to the C-terminus of either fluorescent protein allows macrophage membrane projections to be easily visualized. Scale bars = 10 μm. Expression data representative of more than 50 animals for each construct.

To overcome the limitation in long-term imaging of macrophage/*M*. *marinum* interactions imposed by the loss of *mpeg1* transgene expression, we developed novel transgenic lines based on another of the original four *spi1*-controlled genes, microfibrillar-associated protein 4 (*mfap4*) [37]. The turquoise version of this line has been reported previously by our laboratory [14], but here we describe its characterization and the development of additional tools that expand the repertoire of macrophage-specific tools. To generate an *mfap4* promoter element likely to recapitulate the endogenous, macrophage-specific expression pattern, we amplified ~1.6 kb of genomic sequence at the 5’ end of the *mfap4* locus. Specifically, the promoter element includes 1.5 kb of sequence upstream of the *mfap4* transcription start site, continues through the entire first intron and terminates at the second codon of the second exon (Fig 1A). We elected to retain sequences to direct splicing of the transgene, as mRNA export has been shown to be enhanced following a splicing event [44–46]. In order to ensure translation initiation at the start site of the inserted transgene, we mutated the *mfap4* start codon in the promoter element from ATG to ATT. 5’ RACE indicated a single initiation codon of the endogenous *mfap4* transcript pool, and so alteration of this ATG likely abrogates any translation of any *mfap4*-specific sequence retained at the 3’ end of the promoter element of *mfap4* transgenes (data not shown).

We cloned the amplified promoter as a 5’ Element of the Multisite Gateway Recombination System to facilitate simple, rapid generation of an array of *mfap4*-controlled transgenic constructs through modular assembly of the promoter element with different transgenes [47, 48]. In this manner we generated several independent transgenic zebrafish lines each expressing either the tdTomato (red) [49], dLanYFP (yellow/green) [50], or Turquoise2 (cyan) [51] fluorescent proteins: *Tg(mfap4*:*tdTomato)* ^*xt12*^ and *Tg(mfap4*:*tdTomato-CAAX)* ^*xt6*^, *Tg(mfap4*:*dLanYFP-CAAX)* ^*xt11*^, *and Tg(mfap4*:*Turquoise2)* ^*xt27*^, respectively. Each of these lines exhibited robust expression in cells that appeared to be macrophages (Fig 1B); this is especially apparent for the transgenic lines expressing fluorescent proteins with a C-terminal CAAX motif, resulting in targeting of the transgene to the plasma membrane [42, 43]. In these two lines, long membrane projections characteristic of motile macrophages are always observed (Fig 1Biii and 1Biv). We also observed a modest increase in fluorescence signal in the yolk of two tdTomato-expressing transgenic lines as compared to the yolk of wildtype larvae, but this limited yolk fluorescence was absent in other *mfap4* lines indicating that it is not a general property of the promoter (S1 Fig).

**Fig 2:**
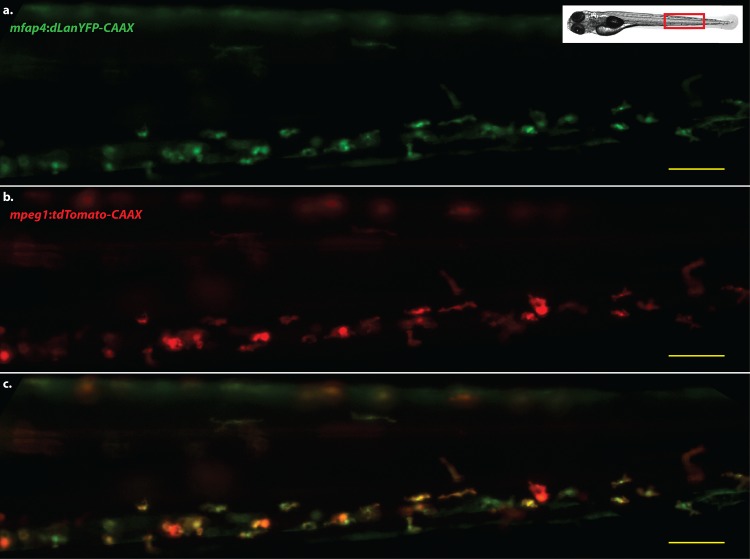
*mfap4* transgene expression colocalizes with the expression of *mpeg1* transgenes. Representative image of the caudal hematopoietic tissue of a double transgenic larva, four days post-fertilization. **a.** Cells expressing dLanYFP via the *Tg(mfap4*:*dLanYFP-CAAX)* ^*xt11*^ transgene. **b.** Cells expressing the tdTomato fluorescent protein via the *Tg(mpeg1*:*tdTomato)* ^*xt3*^ transgene. **c.** Merged image of **a,b**. Scale bars = 50 μm. Images representative of at least 40 animals.

Subsequent populations of these lines derived from stable, germ-line transmitting genomic integrations exhibited transgene expression detectable in a specific population of cells by 36 hours post-fertilization (hpf), consistent with the timing of macrophage development and accumulation of sufficient fluorescent protein to yield a signal [35]. Fluorescence is greatly increased by 48 hpf, and can be detected easily via epifluorescence microscopy at relatively low magnification (e.g., 5x objective lens/0.25 NA). Expression remains robust in the adult and continues to be restricted to cells that appear to be macrophages to at least three months of age, including cells infected with *M*. *marinum* (S2 Fig). Because transgenic lines based on *mpeg1* have been previously characterized as macrophage-specific [6], we sought to determine the specificity of the *mfap4* promoter by crossing *Tg(mfap4*:*dLanYFP-CAAX)* ^*xt11*^ with *Tg(mpeg1*:*tdTomato-CAAX)* ^*xt3*^. We observed essentially complete colocalization of expression of both fluorophores (Fig 2), indicating that *mfap4* transgenics exhibit an expression pattern that is highly restricted to zebrafish macrophages.

**Fig 3:**
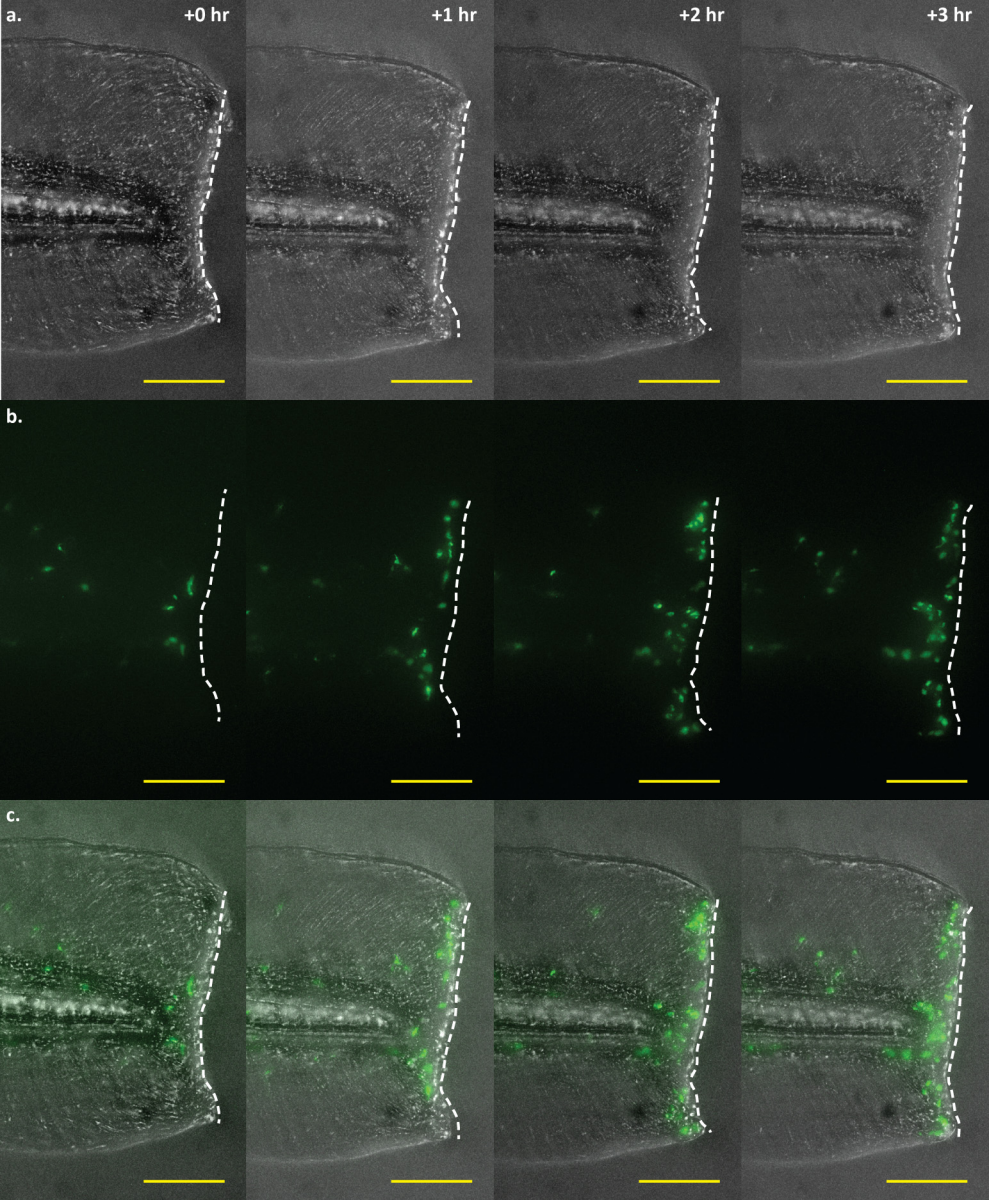
Time-lapse imaging of a tail wound of an *mfap4*:*dLanYFP-CAAX* transgenic larva. Time-lapse imaging of macrophage recruitment to the site of a wound at the posterior end of the larval tail. Macrophage movements may be visualized in real-time via fluorescence imaging of *mfap4* transgenic larvae. Shown here are four representative frames of such an experiment, showing macrophage recruitment to the wound edge at 0, 1, 2, and 3 hours post-wounding. **a.** Brightfield detailing the extent and location of the wound. The wound edge is highlighted as a dashed white line. **b.** Macrophages visualized via dLanYFP fluorescence. The number of cells present proximal to and along the site of the wound can be seen increasing over time as macrophages track toward the damaged tissue. **c.** Merge of brightfield and fluorescence channels. Scale bars = 100 μm. Images representative of 12 animals from two independent biological replicates.

We then sought to demonstrate the utility of *mfap4* transgenics for *in vivo* time-lapse imaging of zebrafish larvae, as well as confirm standard macrophage behaviors of the labeled cell population. We wounded *Tg(mfap4*:*dLanYFP-CAAX)* ^*xt11*^ larvae by excising a small portion of the developing tail fin, and imaged the region over several hours to track the movements of the labeled cell population (Fig 3). Fluorescent cells can be seen tracking toward and accumulating at the edge of the wound, a behavior consistent in both timing and activity for macrophages upon introduction of an inflammatory stimulus [52]. Thus, *mfap4* driven fluorescent proteins and the added CAAX motif do not appear to alter macrophage behavior and, therefore, permit the visualization of membrane projections in migrating macrophages.

### The *mfap4* promoter produces stable transgene expression throughout the course of larval infection with *M*. *marinum*

**Fig 4:**
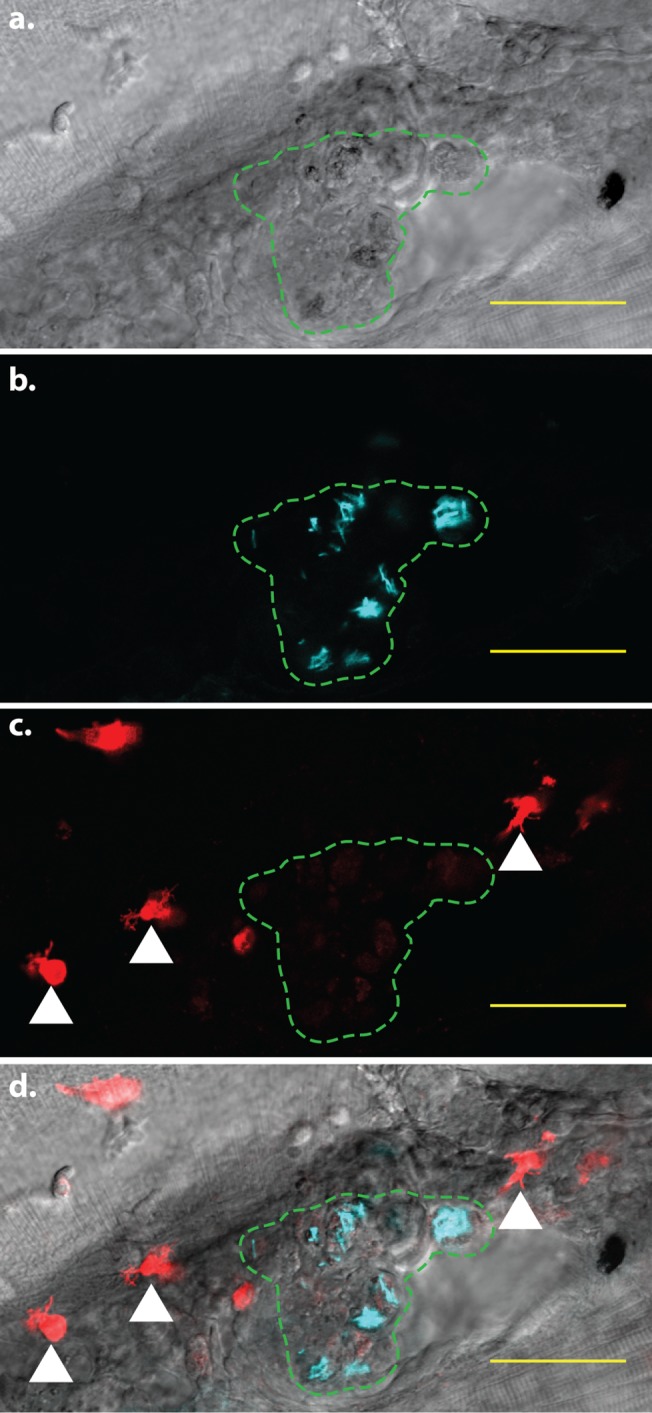
*mpeg1*-mediated fluorophore expression is attenuated in infected cells. Confocal image of a larval granuloma, 5 days post-infection. The region containing infected cells is indicated by the green dashed line. Within each cell, *M*. *marinum* expressing the Cerulean fluorescent protein (cyan signal) are clearly visible. In addition, faint tdTomato fluorescence is visible within the infected cells. White arrowheads indicate examples of bright, uninfected cells exhibiting normal levels of tdTomato fluorescence. **a.** Brightfield detailing the granuloma in which *mpeg1* fluorescent protein expression is attenuated. **b.** Fluorescent *M*. *marinum* in the same area **c.** Macrophages expressing the tdTomato fluorescent protein. **d.** Merged image of **a**-**c**. Scale bars = 50 μm.

The zebrafish larva has become increasingly utilized for performing real-time, *in vivo* analyses of mycobacterial disease pathogenesis within a vertebrate host [14–16]. Such studies are greatly enhanced by the ability to track macrophages during infection, as macrophages are the primary cell type involved in initial interactions with pathogenic mycobacteria [13, 53]. However, it has been previously observed that *mpeg1* expression in zebrafish macrophages becomes attenuated in the presence of *M*. marinum [16], and we also observed a marked reduction in the fluorescence signal within infected macrophages of *mpeg1* transgenic lines, often to the point that many cells within the macrophage population were no longer detectable by fluorescence (Fig 4).

**Fig 5:**
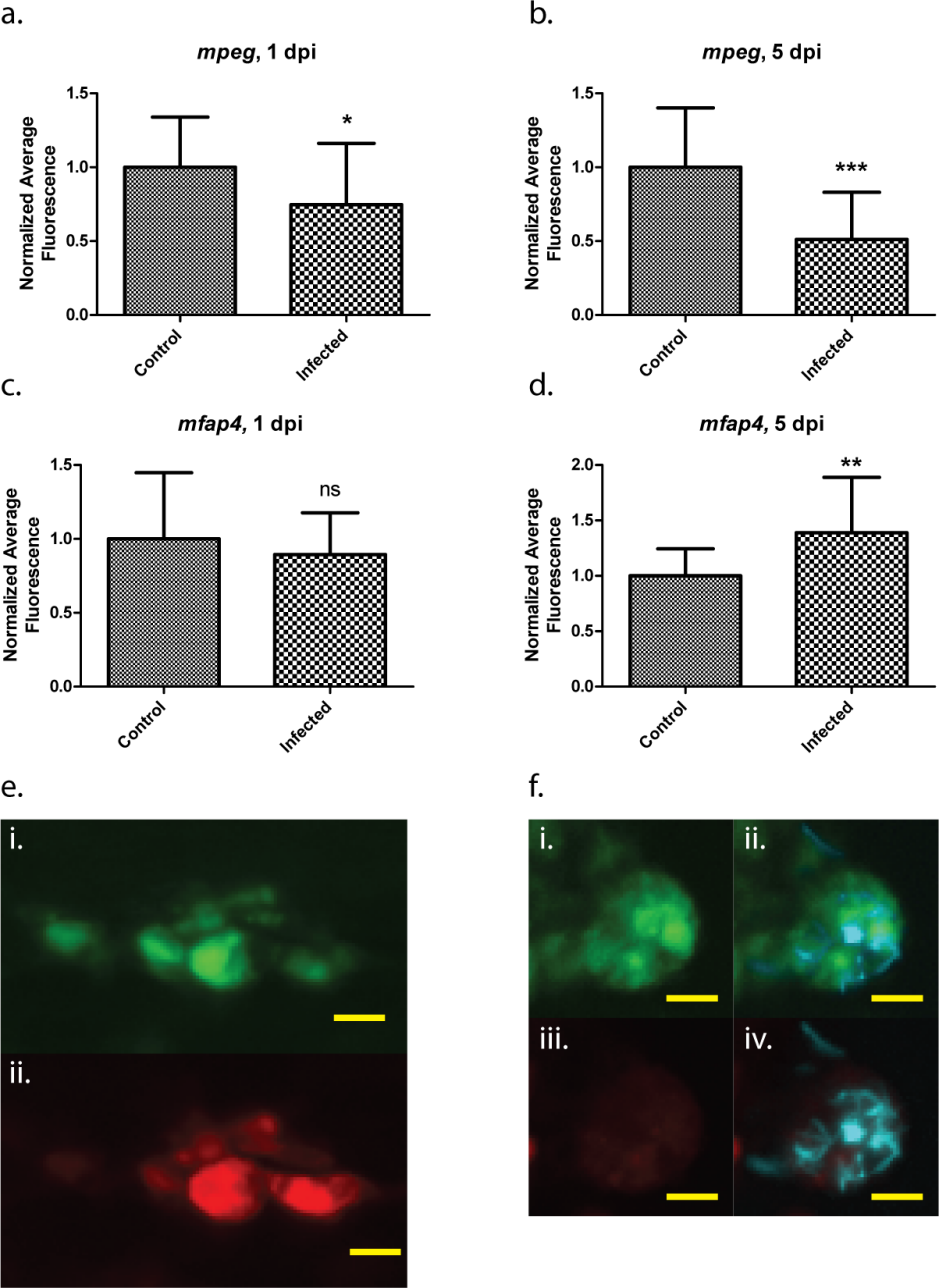
Differential regulation of *mfap4* and *mpeg1* transgenes during mycobacterial infection. Average fluorescence values of either *mpeg1*:*tdTomato-CAAX* or *mfap4*:*dLanYFP-CAAX* fluorescent proteins relative to uninfected, age-matched controls. Five randomly chosen infected macrophages per larva (“Infected”), or five randomly chosen macrophages within uninfected larvae (“Control”) were assessed. 20 larvae were analyzed for each of the infected and control groups at 1 dpi as well as for the Infected group at 5 dpi; 19 larvae were analyzed for the Control group at 5 dpi. Both tdTomato fluorescence (**a,b**) and dLanYFP fluorescence (**c,d**) were measured for the same pool of cells from each larva. **e.** Representative image of *mfap4* transgene expression (i) and *mpeg1* transgene expression (ii) within the same group of cells in an uninfected Control larva, 5 dpi. **f.** Representative image of *mfap4* transgene expression (i) and *mpeg1* transgene expression (iii) within the same group of cells in an Infected larva, 5 dpi; also shown are merged images of fluorescent *M*. *marinum* (cyan signal) with the YFP (ii) and tdTomato (iv) channels. Note the almost total loss of *mpeg1-*mediated fluorescence within the infected group of cells, while *mfap4* transgene fluorescence remains robust. Scale bars = 10 μm. * p < .05, ** p < .005, *** p = .0002. Student’s t-test with Welch’s correction for unequal variances. Error bars indicate +/- SD.

We confirmed via DIC imaging that most of these macrophages were still alive and intact (data not shown), suggesting that expression of the *mpeg1* transgene itself is downregulated by infection. Similar experiments using *mfap4* transgenics, however, suggested that the expression of *mfap4* transgenes was independent of infection status. In order to determine the relative stability of *mfap4* transgene expression in infected zebrafish larvae, the transgenic lines *Tg*(*mpeg1*:*tdTomato-CAAX*)^*xt3*^ and *Tg*(*mfap4*:*dLanYFP-CAAX*)^*xt11*^ were crossed to generate larvae simultaneously expressing both fluorescent proteins in all macrophages. The double transgenic larvae were separated into two groups: the first was infected intravenously with *M*. *marinum* and the other injected with vehicle. Fluorescence intensity of either fluorophore was measured for a randomly-selected subset of infected macrophages from the infected pool, and on macrophages from the same region within the uninfected pool. For both day 1 and day 5 post-infection, the fluorescence intensity of the *mpeg1-*mediated fluorophore was significantly decreased relative to controls, whereas expression of the *mfap4*-mediated fluorophore remained stable and even exhibited a modest increase in expression level at 5 dpi compared to controls (Fig 5). These data include both solitary and granuloma-associated infected macrophages. Notably, infected macrophages in several larvae exhibited barely detectable *mpeg1* transgene fluorescence, while those same macrophages exhibited robust *mfap4* transgene fluorescence in the same cell. The same disparity was not observed among macrophages in uninfected larvae, in which the fluorescence of both fluorophores remained relatively stable. We also observed that expression of either transgene appeared unperturbed in uninfected macrophages within infected larvae, although this was not assessed quantitatively. These data indicate that the altered transgene expression may be largely cell-autonomous, requiring the presence of intracellular *M*. *marinum*.

### The *mfap4*:*iCre*:*p2a-tdTomato* transgenic line allows for macrophage-specific expression of novel and pre-existing Cre-compatible transgenes

**Fig 6:**
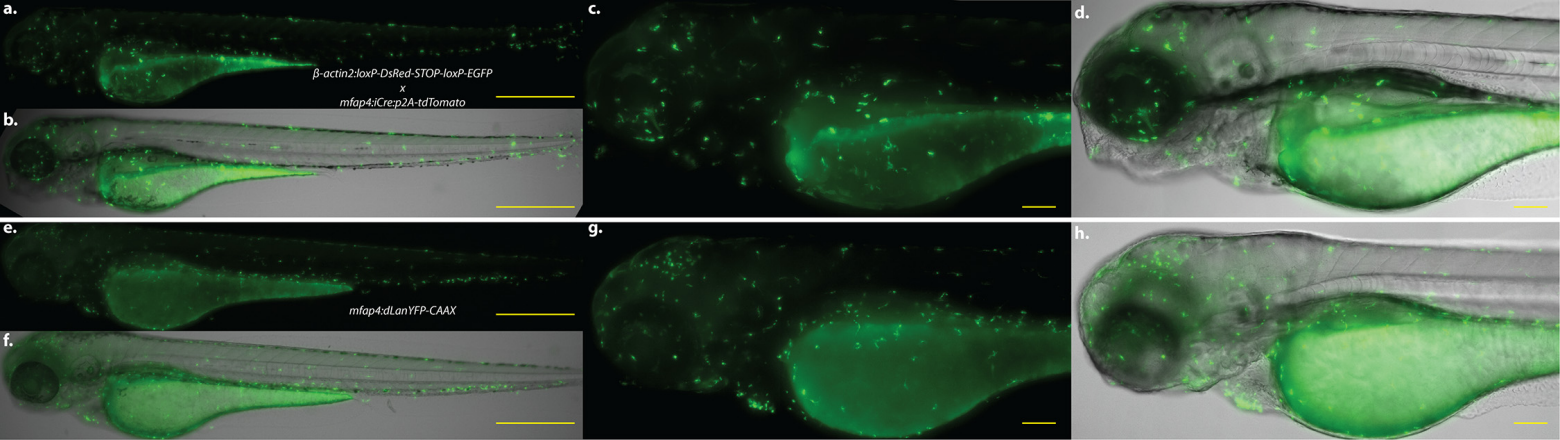
*mfap4*:*iCre*:*p2A-tdTomato* mediates macrophage-specific gene expression of loxP-containing transgenes. **a-d.** Zebrafish larva at 3 dpf expressing both *Tg*(β-*actin2*:*loxP-DsRed-STOP-loxP-EGFP)* ^*s928*^ and *Tg(mfap4*:*iCre*:*p2A-tdTomato)* ^*xt8*^ *;* **a,b.** 50x magnification of GFP fluorescence and merge with brightfield, respectively. **c,d.** 200x magnification of the head, yolk sac, and anterior portion of the gut. **e-h.** Zebrafish larva at 3 dpf expressing *Tg(mfap4*:*dLanYFP-CAAX)* ^*xt11*^ for comparison. **e,f.** 50x magnification of dLanYFP fluorescence and merge with brightfield, respectively. **g,h.** 200x magnification of the head, yolk sac, and anterior portion of the gut. Note that in both larvae, fluorescent protein expression is restricted to macrophages all along the body, with a particular enrichment in the head. Both larvae also exhibit expression within the caudal hematopoietic tissue (CHT). The larvae in **a-d** show a reduced number of fluorescent cells in the nascent macrophage population that predominates in the CHT region, and modestly reduced fluorescent cell population number in regions where mature macrophages have migrated throughout the body. Scale bars for 50x images = 500 μm; scale bars for 200x images = 100 μm.

We have observed that individual transgenes present in zebrafish often exhibit diminishing levels of expression as the transgene design increases in length and complexity (e.g., constructs expressing fluorophore-tagged host proteins, or constructs containing a viral 2A peptide to direct separation of two discrete protein products from a single open reading frame [41]). The lower expression level of longer transcripts is often seen despite the use of stable, lineage-specific promoter elements. To overcome this apparent limitation, as well as to enhance the utility and scope of the *mfap4* promoter, we generated the transgenic line *Tg(mfap4*:*iCre*:*p2A-tdTomato)* ^*xt8*^, resulting in macrophage-specific expression of a codon-optimized Cre recombinase (iCre) [54]; tdTomato serves to identify larvae positive for transgenesis. To assess the efficiency and cell specificity of this system, we crossed the zebrafish line *Tg(*β-*actin2*:*loxP-DsRed-STOP-loxP-EGFP)* ^*s928*^ [55] with *Tg*(*mfap4*:*iCre-p2A-tdTomato)* ^*xt8*^. Progeny that contained both transgenes were identified on the basis of macrophage-specific EGFP expression, indicating successful removal of the DsRed-STOP cassette in macrophages. These larvae thus exhibited strong EGFP expression confined to macrophages but under the control of the zebrafish *β-actin2* promoter (Fig 6). We noted that progeny of this cross have decreased numbers of fluorescent cells, particularly within the CHT region, as compared to *Tg(mfap4*:*dLanYFP-CAAX)* ^*xt11*^ larvae (Fig 6).

## Discussion

These data establish the *mfap4* promoter element as a stable, robust genetic tool for driving macrophage-specific transgene expression in zebrafish. As a component of the Multisite Gateway Recombination system for generating transgenic constructs, this new promoter facilitates the generation of multiple transgenic constructs and subsequent zebrafish lines for which expression is restricted to macrophages. Transgenes driven by the *mfap4* promoter exhibit robust, stable expression independent of larval infection status with *M*. *marinum*, making it particularly useful for the study of interactions between pathogenic mycobacteria and a vertebrate host. Beyond simple visualization of macrophages within zebrafish larvae, transgenes other than fluorescent proteins may be expressed in a macrophage-specific manner, facilitating the study of host factors that are involved in restriction or permissiveness to intracellular mycobacteria.

Using additional genetic tools and common breeding techniques (i.e., *mfap4*-driven Cre recombinase expression), more complex transgenic lines may be generated to further enhance transgene expression in a macrophage-restricted manner. The stronger fluorescent protein expression of these recombined transgenes may be particularly useful for long-term time-lapse imaging requiring high temporal resolution, as much lower exposure times/intensities may be used for each image resulting in significantly less photobleaching and potential phototoxicity. Furthermore, we anticipate that this system will be advantageous for driving macrophage-restricted expression of transgenes designed to rescue or overexpress biologically active proteins.

We note that the *mfap4*:*iCre* transgenic line led to strong expression in macrophages, but overall produced a reduced number of labeled macrophages in the CHT region. Because the CHT is the major site of myelopoiesis at this age, this disparity may reflect a delay in the accumulation of Cre recombinase in nascent macrophages. Alternatively, the efficiency of Cre-mediated recombination for the partner transgene tested may be less than 100 percent.

Overall, we anticipate that this promoter element as well as the repertoire of macrophage-specific transgenic lines we have established will be useful in the study of the vertebrate innate immune system. In particular, stable expression from *mfap4* transgenes during infection will enable long-term imaging and manipulation of macrophages as they interact with intracellular pathogens. In addition, these transgenes should be compatible with FACS [37, 56] of macrophage populations, both uninfected and infected, from whole animals, facilitating future studies into the modulation of the macrophage transcriptome either by pathogen or host during infection with a variety of pathogens.

## Supporting Information

S1 Fig Whole animal expression pattern of *mfap4* transgenic lines. All larvae shown are 3 dpf. Each panel pairs a representative transgenic animal with a non-transgenic animal to provide a direct comparison of background fluorescence in the relevant excitation/emission channel. **a.** *Tg(mfap4*:*tdTomato)* ^*xt12*^. **b.** *Tg*(β-*actin2*:*loxP-DsRed-STOP-loxP-EGFP)* ^*s928*^ X *Tg(mfap4*:*iCre*:*p2A-tdTomato)* ^*xt8*^. **c.** *Tg*(*mfap4*:*dLanYFP-CAAX*)^*xt11*^. **d.** *Tg(mfap4*:*tdTomato-CAAX)* ^*xt6*^. Scale bars = 500 μm.
(TIF)

S2 Fig *mfap4* transgenes are robustly expressed in adult zebrafish macrophages. **a.** Frozen 20 μm section of 3 month old adult *Tg(mfap4*:*tdTomato-CAAX)* ^*xt6*^ zebrafish liver tissue, 2 weeks post-infection (wpi) with approximately 400 c.f.u. of *M*. *marinum* expressing the Cerulean (cyan) fluorescent protein. i. Macrophages expressing tdTomato. The stellate morphology expected of Kupffer cells, specialized resident macrophages of the liver, can be seen. ii. *M*. *marinum*. iii. DAPI. iv. Merge of i-iii. **b.** Frozen 20 μm section of kidney tissue from the same fish. i. Macrophages expressing tdTomato. ii. *M*. *marinum*. iii. DAPI. iv. Merge of i-iii. For both **a** and **b**, tdTomato (macrophage) and Cerulean (*M*. *marinum*) fluorescence signals are derived directly from fixed fluorescent protein; other than DAPI, no additional staining was performed. Scale bars = 50 μm.
(TIF)
