# Supplementary figures and images for: The Macrophage-Specific Promoter mfap4 Allows Live, Long-Term Analysis of Macrophage Behavior during Mycobacterial Infection in Zebrafish
Source: PLoS One. 2015 Oct 7;10(10):e0138949. doi: 10.1371/journal.pone.0138949 (PMC4596833; doi:10.1371/journal.pone.0138949)

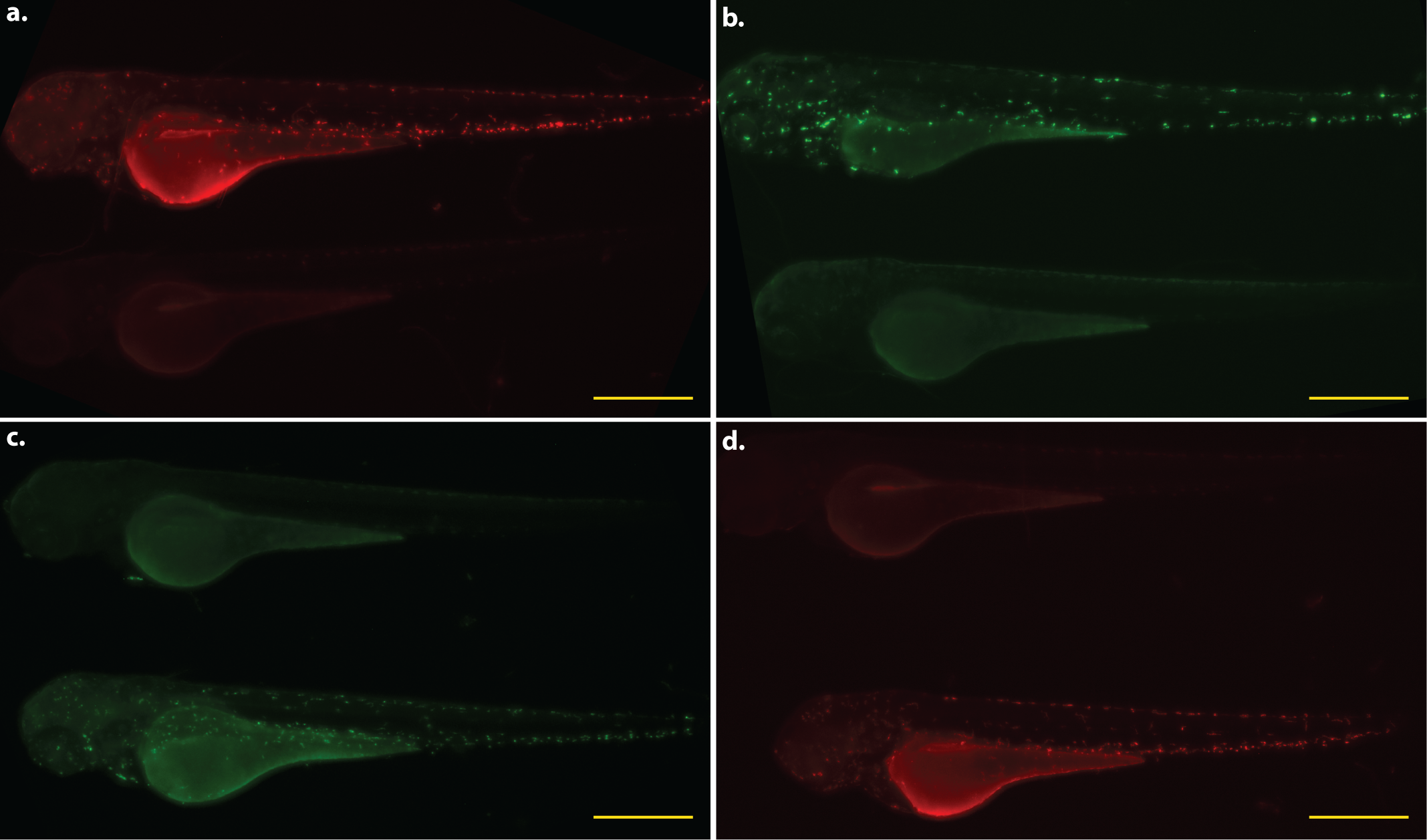

Supplement: S1 Fig — All larvae shown are 3 dpf. Each panel pairs a representative transgenic animal with a non-transgenic animal to provide a direct comparison of background fluorescence in the relevant excitation/emission channel. a. Tg(mfap4:tdTomato) xt12. b. Tg(β-actin2:loxP-DsRed-STOP-loxP-EGFP) s928 X Tg(mfap4:iCre:p2A-tdTomato) xt8. c. Tg(mfap4:dLanYFP-CAAX)xt11. d. Tg(mfap4:tdTomato-CAAX) xt6. Scale bars = 500 μm. (TIF) [file pone.0138949.s001.tif]

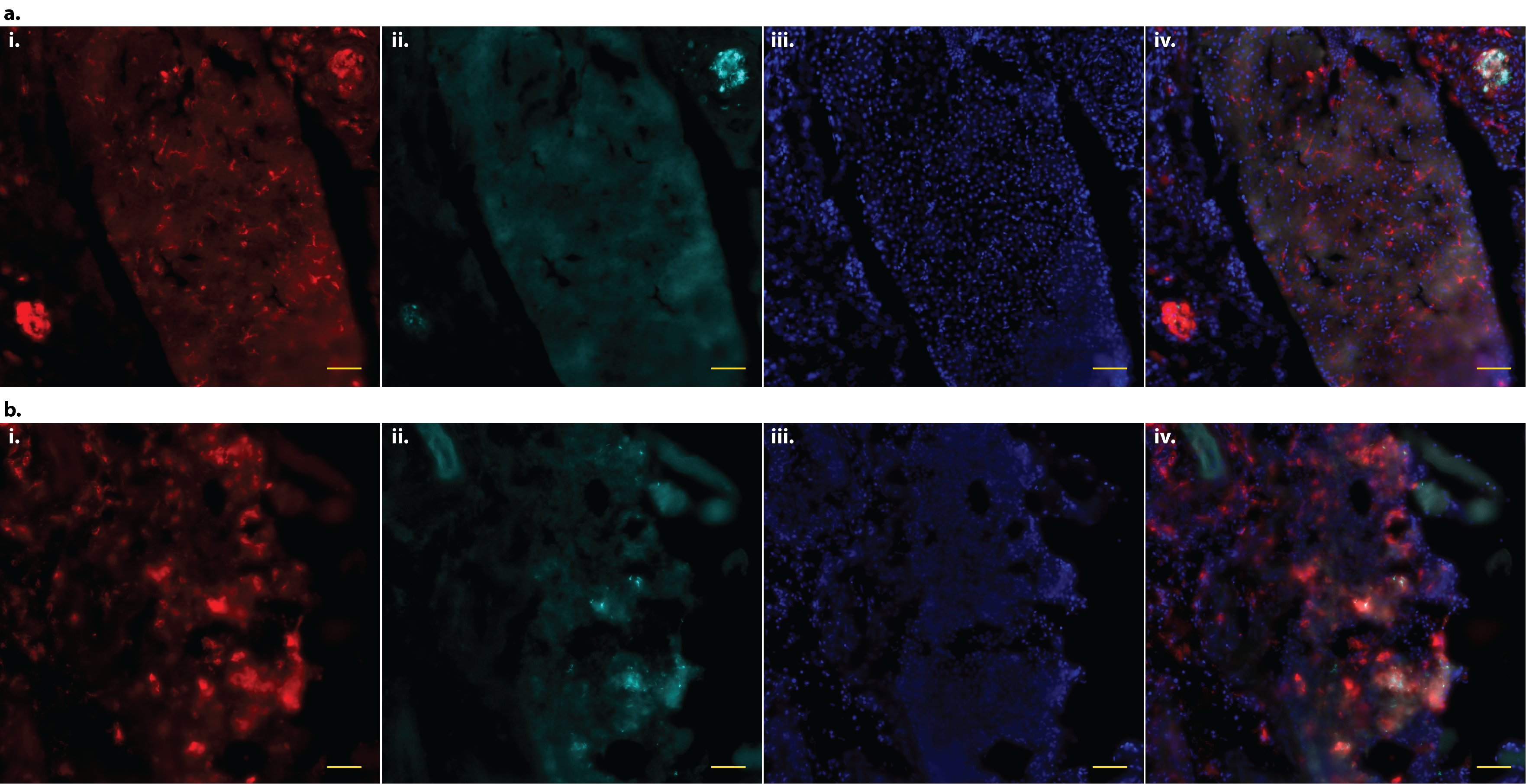

Supplement: S2 Fig — a. Frozen 20 μm section of 3 month old adult Tg(mfap4:tdTomato-CAAX) xt6 zebrafish liver tissue, 2 weeks post-infection (wpi) with approximately 400 c.f.u. of M. marinum expressing the Cerulean (cyan) fluorescent protein. i. Macrophages expressing tdTomato. The stellate morphology expected of Kupffer cells, specialized resident macrophages of the liver, can be seen. ii. M. marinum. iii. DAPI. iv. Merge of i-iii. b. Frozen 20 μm section of kidney tissue from the same fish. i. Macrophages expressing tdTomato. ii. M. marinum. iii. DAPI. iv. Merge of i-iii. For both a and b, tdTomato (macrophage) and Cerulean (M. marinum) fluorescence signals are derived directly from fixed fluorescent protein; other than DAPI, no additional staining was performed. Scale bars = 50 μm. (TIF) [file pone.0138949.s002.tif]
